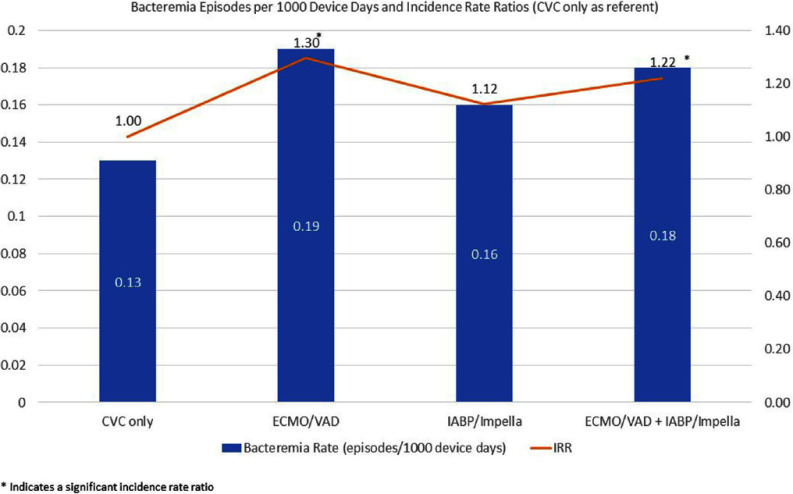# Infection on the Sidelines: Evaluating Bacteremia Rates in Device-Dependent Cardiology Patients

**DOI:** 10.1017/ash.2025.284

**Published:** 2025-09-24

**Authors:** Jessica Seidelman, Heather Pena, Alexandria Hunt, Brittain Wood, Polly Padgette, Erin Gettler, Jeffrey Keenan, Deverick Anderson, Becky Smith

**Affiliations:** 1 Duke University; 2 Duke University Hospital; 3 Duke University; 4 Duke; 5 Duke University Medical Center; 6 Duke Center for Antimicrobial Stewardship and Infection Prevention; 7 Duke University Medical Center

## Abstract

**Introduction:** Patients with mechanical circulatory support (MCS) devices, such as ventricular assist devices (VAD) and extracorporeal membrane oxygenation (ECMO), are excluded from the National Healthcare Safety Network (NHSN) central line-associated bloodstream infection (CLABSI) criteria, whereas patients with intra-aortic balloon pumps (IABP) and Impella devices remain included. Since both MCS and Impella/IABP devices are associated with bloodstream infection risks, this study compares bacteremia rates among patients with VAD/ECMO, IABP/Impella, and central venous catheters (CVCs) to inform more accurate infection reporting. **Methods:** Using a surveillance database, we retrospectively reviewed bloodstream infections among patients with a CVC, ECMO/VAD, or IABP/Impella admitted to Duke University Hospital Cardiology units from January 2019 to July 2024. Bacteremia episodes were calculated per 1000 device days, with de-identified data pooled for final analysis. **Results:** A total of 849 bacteremia episodes were observed in patients with only a CVC (0.14 episodes/1000 device days), 98 in patients with ECMO/VAD (0.19/1000 device days), and 64 in patients with IABP/Impella (0.16/1000 device days). (Figure 1) Bacteremia incidence rate ratio (IRR) in patients with ECMO/VAD compared to patients with only a CVC was 1.30 (95% CI 1.05, 1.60, p-value 0.01). Bacteremia IRR in patients with Impella/IABP compared to patients with only a CVC was 1.12 (95% CI 0.87, 1.45, p-value 0.37). However, when we combined both ECMO/VAD and IABP/Impella bacteremia episodes and compared the bacteremia rate to patients with only a CVC, the incidence rate ratio was 1.22 (95% CI 1.03, 1.44, p-value 0.02). **Discussion:** The significantly different combined bacteremia rates among ECMO/VAD and IABP/Impella suggest that both device categories have significantly higher rates of bacteremia compared to CVC-only patients. Thus, NHSN should reconsider NHSN exclusion criteria for Impella/IABP patients similar to that for ECMO/VAD patients. Further collaboration with institutions, could strengthen findings and refine infection control protocols in high-risk, device-dependent patients.

**Figure:**